# “Born to Run”? Not Necessarily: Species and Trait Bias in Persistent Free-Living Transgenic Plants

**DOI:** 10.3389/fbioe.2018.00088

**Published:** 2018-07-03

**Authors:** Norman C. Ellstrand

**Affiliations:** Department of Botany and Plant Sciences, University of California, Riverside, Riverside, CA, United States

**Keywords:** dispersal, engineered genes, feral plants, unmanaged populations, pollen gene flow, seed gene flow, seed spillage, volunteers

## Abstract

The possibility of transgenes from engineered plants ending up in unmanaged populations with undesirable consequences has been a long-term biosafety concern. Experience with traditionally improved plants reveals that most cases of such gene escape have been of little consequence, but on occasion they have led to the evolution of problematic plants or have resulted in an increased extinction risk for wild taxa. Three decades have passed since the first environmental release of transgenic plants, and more than two decades since their first commercialization. Examples of transgenes gone astray are increasingly commonplace. Transgenic individuals have been identified in more than a thousand free-living plant populations. Here I review 14 well-documented consolidated “cases” in which transgenes have found their way into free-living plant populations. Some as transient volunteers; others appear to be persistent transgenic populations. The species involved in the latter are not representative of the current commercialized transgenic crops as whole. They tend to share certain traits that are absent or rare in the transgenic crops that do not exist as persistent populations. The traits commonly occurring in species with persistent transgenic free-living populations are the following, in descending order of importance: (1) a history of occurring as non-transgenic free-living plants, (2) fruits fully or partially shattering prior to harvest, (3) have small or otherwise easily dispersed seeds, either spontaneously or by seed spillage along the supply chain from harvest to consumer, (4) ability to disperse viable pollen, especially to a kilometer or more, (5) perennial habit, and (6) the transgene's fitness effects in the recipient environment are beneficial or neutral. Based on these observations, a thought experiment posits which species might be the next to be reported to occur as free-living transgenic populations.

## Introduction

An early concern regarding genetically engineered plants was that the unintended movement of transgenes by seed, pollen, or even individuals might have undesirable consequences. The initial focus was that spontaneous hybridization between a transgenic crop and a nearby wild or weedy relative would result in the evolution of a new plant pest (Colwell et al., 1985; National Research Council, 1989). Goodman and Newell (1985) summarized the concern succinctly: “The sexual transfer of genes to a weedy species to create a more persistent weed is probably the greatest environmental risk of planting a new variety of crop species” noting that the risk is not necessarily restricted to transgenic varieties. Indeed, even though spontaneous hybridization between non-transgenic crops and wild plants is usually of little consequence, in a few cases, such hybridization has had economically disastrous consequences, such as the evolution of Europe's weed beet (Ellstrand, 2003) and Brazil's herbicide-resistant weedy rice (Merotto et al., 2016). Experience from traditionally improved crops has demonstrated that wandering crop genes can have other negative environmental effects. For example, crop-wild hybridization between the domesticated coconut palm and its wild ancestor has resulted in the extinction of the latter (Ellstrand, 2003).

The unintended movement of crop genes was an agronomic problem long before plants were genetically engineered. The primary problems were associated with intervarietal mixing via pollen or seed. Immigrant gene flow by pollen from cross-compatible plants outside of a breeder's selection plots (“pollen contamination”) would result in seeds sired by non-experimental plants and frustrate plant improvement efforts. Imagine the offspring from unexpected cross-pollination between backyard pumpkins and a breeder's yellow crooked-neck squash. Consequently, breeders attempt to spatially isolate their experimental plots as well as their seed multiplication fields from possible sources of unwanted pollen (Kelly and George, 1998). Cross-pollination is not the only cause of unintended genetic admixture. Segregation strategies are necessary to prevent accidental mixing of seeds of different commercial varieties (“seed contamination”) to maintain varietal purity (identity preservation) for the consumer, Strayer, 2002). To illustrate, a farmer intending to grow sweet corn would be disappointed to find that 20 percent of her plants were a popcorn variety.

With the recognition that 100% genetic purity is difficult or impossible to obtain, acceptable thresholds of unintended genetic material have been standardized for different crops and their purposes. For example, the Organization for Economic Co-operation and Development (OECD) Seed Scheme requires a minimum of 99.7% varietal purity for oilseed groundnut (aka peanut) to be used for basic seed (seed used as the basis for varietal seed increase) and reduces the requirement to 99.5% for certified seed, the purest type of seed normally grown by commercial farmers (OECD, 2018). Generally, farmers and others who deal commercially with crops and crop products anticipate and tolerate low levels of genetic mixing. Given the vigilance of the seed industry to minimize contamination, low levels of within-crop varietal seed admixture have rarely caused substantial harm.

Regulators recognize gene flow in their decision-making and that it is sometimes likely to occur. Therefore, the consideration of transgene flow and its consequences is a standard component of national regulatory risk assessment. For example, in the United States, transgenic plants are allowed to be grown under Notification or Permit only if the applicant describes methods of preventing gene flow (National Research Council, 2002). Likewise, the deregulation in the United States considers the impacts of possible varietal intermixing as well as the establishment of the transgene in free-living populations. Potential gene flow impacts have sometimes led to controversy during the deregulation process in the United States. Such controversy catalyzed the requirement for the United States Department of Agriculture to conduct appropriate studies and create an Environmental Impact Statement for certain regulated articles to be deregulated. Note that such assessments are conducted only for the country involved in the regulatory decision. It is not necessary and perhaps improper, for, say, the United States regulators to make a judgment about the environmental impacts of a transgenic crop in its center of origin (e.g., maize in Mexico) (National Research Council, 2002).

Thus, it is not surprising that unintentional intervarietal mixing by seed or cross-pollination involving transgenic cultivars is not uncommon. Furthermore, the extraordinary sensitivity of polymerase chain reaction–based techniques allows the detection of transgenes at extraordinarily low frequencies (Demeke et al., 2006) The unintended occurrence of transgenes or a transgenic variety is increasingly characterized by the terms “adventitious presence” (Kershen and McHughen, 2005; Demeke et al., 2006; Council for Agricultural Science Technology, 2007), and “low level presence” (Stein and Rodríguez-Cerezo, 2010; Smyth et al., 2017) as alternatives to “contamination,” perhaps because the latter carries negative connotations in other contexts. The two new terms are often used interchangeably. However, while adventitious presence of transgenes often occurs at a “low level,” but it does not necessarily require that the frequency of the unexpected genetic material be “low.”

Reports of transgenes out-of-place have steadily accumulated (Price and Cotter, 2014) since the commercialization of transgenic crops in the mid-1990s. These reports frequently attract the attention of the popular press (e.g., Ledford, 2007). A few scholarly reviews have inventoried the many heterogeneous cases of transgenes in a wide variety of unintended venues (Ellstrand, 2012; Bauer-Panskus et al., 2013; Ryffel, 2014). Those reviews focus on the examples of transgene flow, transgene flow's potential consequences, and improved containment. But none have focused on the biology of the species and traits involved in free-living populations as lessons for environmental biosafety risk assessment.

Initially, the assumption was that all crop transgenes would end up in free-living populations. More than a decade ago, Marvier and Van Acker (2005) stated “the movement of transgenes beyond their intended destinations is a virtual certainty.” It is a good times to test that hypothesis. A handful of transgenic crops have been planted in ever increasing acreage for two decades. If “movement beyond intended destinations” is a virtual certainty, that most widely planted transgenes should have all moved beyond their intended destinations by now. The data presented in the earlier reviews suggests that this is not the case.

In particular, to my knowledge, the following questions have not been addressed: Is there anything biologically different about those species and their transgenic traits that set them apart from the species that have not had their transgenes establish on their own? Have any of the free-living populations created environmental/agronomic problems? Are there any biological correlates for those cases? Given that both genetic engineering and gene editing are likely to soon lead to an accelerating number of products involving of a proliferation of improved species based on an abundance of novel traits, the ability to separate escape-prone trait-species combinations from others should expedite risk assessment in a way similar to the “tiered” approach offered by a recent National Research Council (2017) report.

Here I review the free-living plant populations (volunteer, feral, weedy, and wild) that have been found to have transgenic individuals. I identify which have created environmental (agronomic or otherwise) problems. I focus on the biology of species that have established persistent (multiyear) populations to identify any commonalities. I conclude with a crude model for predicting the kinds of plants with novel traits that might establish free-living populations in the future.

## Materials and methods

Incidents of plant transgenes in unintended situations are now too numerous to inventory individually. Thus, I sought and organized “cases” that represent specific combinations of plant species, transgene regulatory status at discovery, and occurrence type. Cases were collected from pre-existing reviews (Ellstrand, 2012; Bauer-Panskus et al., 2013; Ryffel, 2014) and supplemented with a literature review, with special attention to incidents not covered in the previous reviews. Despite that effort, the review is not necessarily exhaustive. Some cases are too poorly studied or documented to report here. Here the concentration is on the most convincing and informative examples. Given the frequent of such reports, I anticipate that new examples will be reported before this article reaches publication. All the cases selected are substantiated by peer-reviewed scholarly articles and/or government publications.

I used the following criteria for choosing the cases:

1. Plants sampled were free-living. That is, sampled plants were growing without intentional human management to promote their survival and growth. Free-living plants can be volunteers, feral individuals, weeds, or wild plants. Unmanaged sites may or may not be human-disturbed. Free-living plants can also be growing within a planted crop. Judging whether or not free-living populations are persistent is often challenging without multi-year study or genetic analysis. Indeed, populations of roadside volunteer plants that are constantly replenished by seed spilled from transport vehicles persist and are considered persistent here. They are analogous to natural wild populations in marginal sites maintained by natural seed rain from nearby robust populations of the same species (Harper, 1977).
2. One or more individuals containing at least one transgene are identified from the population.
3. The transgene is confirmed by more than its gross phenotype. For example, plants surviving a glyphosate application must have a transgene or its product be confirmed by immunochemical or DNA-based analysis.

To maintain a focused scope, the following, somewhat idiosyncratic, categories of transgenes out-of-place were not considered for this review:

1. Grain or seed mixed with adventitious transgenic grain approved for cultivation in the region grown (e.g., Booker et al., 2014).
2. Transgenic plants, grain, or seed not at all approved for cultivation that is mistakenly cultivated, but not does not occur in free-living populations (e.g., USDA, 2007, 2017; Clapp, 2008; Dyer et al., 2009; Bashandy and Teeri, 2017).
3. Grain or seed with transgenes approved for cultivation in the country of origin but unintentionally distributed to countries in which it is not approved but is not known as free-living individuals in such countries. This criterion excludes the notorious “Starlink” affair (see box 2.1 in National Research Council, 2004) and other cases (e.g., University of California Davis, 2003).
4. Intentionally cultivated transgenic crops unapproved for cultivation in the region grown (that is, intentional illegal cultivation, such as transgenic soy in Brazil prior to its formal approval (Daroit and Nascimento, 2009); and
5. Spontaneous interspecific or intervarietal seed produced involving a transgenic parent but not resulting in any free-living plants (e.g., Zapiola and Mallory-Smith, 2012).
6. Coexistence issues (e.g., Devos et al., 2009), that is unintended successful paternal fertilization (gene flow by pollen) by a transgenic crop into a crop grown for a market that is intended to be free of materials from transgenic plants (such as plants grown organically in the United States; Manshardt et al., 2007).
7. Cases of non-compliance involving “alleged” volunteers (e.g., www.aphis.usda.gov/aphis/ourfocus/biotechnology/sa_compliance_and_inspections/ct_compliance_history).

The foregoing excluded cases are interesting and important in their own right but fall beyond the scope of this review.

## Results and discussion

Table 1 summarizes 14 cases of transgenes occurring in free-living populations. Altogether, they represent more than a thousand transgenic populations that are the result of dozens of dispersal incidents. The majority of the 14 cases involve multiple plants and populations. One case involves a single transgenic interspecies hybrid individual.

**Table 1:** Free-living^a^ populations containing transgenic plants.

**Species (common name)**	**Nature and location of transgenic plants**	**Transgene: trait^b^, regulatory status^c^**	**Probable process of transgene origin**	**Current environmental or agronomic impact ?**	**Relevant citation(s)**
*Agrostis stolonlifera* (creeping bentgrass)	Increasing numbers (hundreds) of glyphosate tolerant plants in the US state of Oregon in ca. 100 sq. km. sampled area. Persistent and spreading.	GT^*^	Initially, spontaneous seed and pollen dispersal from field test sites in Oregon. Subsequent pollen-mediated gene flow spreads the transgene.	Yes, weed of irrigation canals.	(Zapiola et al., 2008; Zapiola and Mallory-Smith, 2017) and references therein
*A. stolonilfera* (creeping bentgrass) *x. A. gigantea* (redtop)	Infrequent transgenic hybrid individuals in the US state of Oregon	GT^*^	Spontaneous hybridization with transgenic *A. stolonilfera* grown under “field trial” conditions as male parent or transgenic free-living plants described in the entry above	No	(Zapiola and Mallory-Smith, 2017)
*B. napus* (Argentine oilseed rape)	Hundreds of largely persistent populations along grain transport lines in Japan (> 400) and Switzerland (13), both of which prohibit cultivation of transgenic oilseed rape. Multi-year studies show frequency of transgenics varies over time and space	GT^+^,gT^+^	Repeated seed spillage during grain transport from ports and international borders to processing facilities. Subsequent cross-pollination among varieties gives rise to multiple herbicide tolerance	No.	(Hecht et al., 2014); (Katsuta et al., 2015) and references therein
*B. napus* (Argentine oilseed rape)	Most plants in several persistent populations in three separate cultivated and non-cultivated areas in Buenos Aires Province, Argentina	GT^+^	Unknown, no recent records of oilseed rape production in those locations.	Unknown. (Alternative herbicides are available to control this localized agronomic weed.)	Pandolfo et al., 2016
*B. napus* (Argentine oilseed rape)	Hundreds of volunteer and persistent feral populations in agronomic and ruderal environments in countries in which transgenic canola is cultivated: Canada (provinces of Alberta, British Columbia, Manitoba, and Saskatchewan), U.S. (states of California and North Dakota), and the Australian state of Western Australia	GT, gT	Spontaneous seed dispersal prior to harvest. Seed spillage during grain transport. Subsequent cross-pollination among varieties gives rise to multiple herbicide tolerance.	Yes. “Volunteer” oilseed rape has become an important agricultural weed of Manitoba and elsewhere in Canada. Evolution of multiple herbicide tolerance through cross-pollination makes control more challenging.	Hall et al., 2000; Yoshimura et al., 2006; Knispel et al., 2008; Knispel and McLachlan, 2010; Schafer et al., 2011; Munier et al, 2012; Busi and Powles, 2016;
*B. napus* (Argentine oilseed rape) x *B. rapa* (wild birdsrape)	2 transgenic wild x crop hybrid swarms adjacent to cultivated oilseed rape in Québec, Canada. Present for multiple years. When last sampled, transgene frequency decreasing	GT	Spontaneous hybridization with crop as pollen parent.	No.	Warwick et al., 2003, 2008
*B. napus* (Argentine oilseed rape) x *B. rapa* (wild birdsrape)	Single plant on a Vancouver roadside far from a *B. rapa* population	GT	Unknown	No.	Yoshimura et al., 2006
*B. rapa* (Polish oilseed rape; domesticated birdsrape)	3 persistent populations in cultivated and disturbed sites in Buenos Aires Province, Argentina; persisting for >4 years	GT^+^	Unknown	Unknown. (Alternative herbicides are available to control this localized agronomic weed.)	Pandolfo et al., 2018
*Carica papaya* (papaya)	22% of feral plants sampled from Oahu and Hawai'i islands of the U.S. state of Hawai'l	VR	Spontaneous gene flow by seed and pollen from cultivated plantations.	No	Manshardt et al., 2016
*Gossypium hirsutum* (cotton)	Transgenic feral/wild plants identified from multiple sites in each of 4 regions of Mexico. Genetic evidence of multigeneration persistence.	GT,LR	Seed spillage from vehicles carrying post-milled feed grain with some subsequent spontaneous intermating	No	Wegier et al., 2011
*Medicago sativa* (alfalfa)	Of 404 roadside sites with feral alfalfa plants in the US states of California, Idaho, and Washington, over 100 contained some transgenic plants. Genetic evidence of persistence.	GT	Seed spillage from transports and pollen flow from nearby alfalfa seed and hay production fields with subsequent intermating.	No	Greene et al., 2015
*Triticum aestivum* (bread wheat)	Modest numbers of plants in a single agricultural field in each of the US states of Oregon (2013), Montana (2014), and Washington (2016). Each incident represents a different transgenic event	GT^*^	Unknown; each incident seems to be independent of the others.	No. (However, USDA APHIS BRS has changed the field trial application method for transgenic wheat from the notification process to the more stringent permit process)	USDA, 2014, 2015, 2016
*Zea mays* (maize)	In 2002 and-2004 regulatory inspectors found volunteer transgenic maize engineered to express industrial (including pharmaceutical) compounds growing in soybean, sorghum, and fallow fields in the US states of Nebraska and Iowa	IC^*^	The volunteers germinated from dormant seed in the soil from the previous year's field trial.	Eradicated.	National Research Council, 2017 (see Box 3.2)
*Zea mays* (maize)	Extensive sampling over several years finds only modest numbers of transgenic volunteers in the general vicinity of grain-handling ports of South Korea, a country in which cultivation of such plants is prohibited	GT^+^, gt^+^, LR^+^	Seed spillage during grain transport from ports	No	Lee et al., 2009; Park et al., 2009 (Han et al., 2014) and references therein

a *“Free-living” signifies populations that occur without intentional human intervention including plants that are volunteers, ferals, weeds, and wild individuals*.

b GT, glyphosate (herbicide)-tolerant; gT, glufosinate (herbicide) tolerant; LR, lepidopteran-resistant; VR; virus resistant; IC expression of industrial compounds

c *No superscript means, “Deregulated in that country or countries at the time of discovery”. Otherwise*:

* *not authorized for environmental release globally at time of discovery*;

+ *not authorized for environmental release at time and place of discovery*.

In many cases, the probable dispersal incidents are well-known. In some of those cases, the transgene had entered pre-existing established free-living populations. In others, the transgenic plants themselves appear to be volunteers or the founders of new transgenic populations. And in a few cases, it is difficult to determine their precise origins.

Contrary to initial concerns, crop transgenes have moved into truly wild populations in only a minority of cases. Four entries in Table 1 detail movement of transgenes into the wild: a herbicide tolerance event from oilseed rape into two populations of wild birdrape in Quebec, Canada (Warwick et al., 2003, 2008), an herbicide tolerance event from creeping bentgrass field trials into wild populations of the same species and of a congener in Oregon, USA (Zapiola and Mallory-Smith, 2017), as well as and herbicide-tolerant and lepidopteran-resistant events in cultivated cotton into weedy-wild populations of the same species (Wegier et al., 2011).

Seed dispersal, anthropogenic or spontaneous, is a common component of many of those cases. Seed spillage from grain transport appears to have played a major role in the naturalization of transgenic feral Argentine oilseed rape populations (*Brassica napus*) both in countries where it is cultivated and in countries where it is imported but prohibited from cultivation. Seed spillage plays a similar role for feral transgenic alfalfa populations in the United States and feral transgenic cotton populations in Mexico as well as a modest number of volunteer transgenic maize plants near ports in South Korea. In contrast, spontaneous seed and pollen dispersal events from a set of field trials account for the establishment and spread of transgenic creeping bentgrass in the US state of Oregon and beyond.

In a few cases, spontaneous pollen flow alone accounts for the evolution of crop-wild hybrids. Spontaneous pollen flow and subsequent pre-harvest shattering played key roles in the evolution multiple transgenic herbicide tolerant oilseed rape in Canada (Hall et al., 2000). But, for a greater fraction of the cases, the primary role of spontaneous pollen flow in transgene spread involves cross-pollination among seed dispersed colonists as well as cross-pollination with pre-existing non-transgenic feral plants of that species.

The regulatory status at the time of discovery varies among cases. A substantial minority (5 cases) involve free-living transgenic populations in regions where that crop had been approved for cultivation: alfalfa, Argentine oilseed rape, cotton, and papaya. These situations are of little surprise. The remainder of the cases were equally split between crops whose environmental release were prohibited worldwide at the time of discovery and those whose environmental release was prohibited at the site of discovery but not globally.

Let's return to the hypothesis that “the movement of transgenes beyond their intended destinations is a virtual certainty” (Marvier and Van Acker, 2005) and examine how it holds up when the unintended destinations are free-living populations. A handful of transgenic crop species have been planted in ever increasing acreage for almost two and a half decades: mostly notably maize, soybean, and cotton, but also oilseed rape, papaya, and squash. If “movement beyond intended destinations” is a virtual certainty, those should have all moved by now.

Have they? For certain long-standing transgenic crops, the answer is yes. There are good numbers of free-living populations of papaya, cotton, and (especially) Argentine oilseed rape. But long-standing transgenic soybean has yet to volunteer or go feral (and not without monitoring for its escape; e.g., Lee et al., 2009). Its transgenic partner, maize, has only occurred as first-and-last-generation volunteers in a tiny number of cases. For these two crops the virtual certainty of establishment has not been realized, possibly because they have been handicapped for free-living by their particular history of domestication (Owen, 2005). Another old-timer that has stayed on the farm is transgenic virus resistant squash (*Cucurbita pepo*). Although free-living transgenes have been sought in free-living populations of *C. pepo*, they have not been found (Prendeville et al., 2012).

In contrast, two newcomers have rapidly established feral transgenic populations, one of them more than a decade before deregulation. In the case of transgenic creeping bentgrass, pollen flow and a single localized wind event helped the transgene migrate from a set of field trials and establish in dozens of unmanaged sites. In the case of alfalfa, the feral populations are not far from transgenic alfalfa production areas and seed transport lines. The other relatively recently commercialized transgenic crop, sugar beet, has not been involved with the establishment of free-living populations or even resulted in unwanted volunteers at this time.

Thus, the “virtual certainty” seems more certain for certain species. Let's examine the crops in Table 1 that have the predilection for itinerant trangenes (excluding those known only as volunteers, maize and wheat): alfalfa, oilseed rape (Argentine and Polish), cotton, creeping bentgrass, and papaya—and compare them to the old-timer transgenic crops that prefer to stay at home. Four of the five wandering crops are multi-year perennials. The other major commercial transgenic crops—maize, soy, squash, sugar beet, are not.

Seed dispersal appears to play the most important role in establishing free-living populations. Spillage from transports creates a regular seed rain on the sides of roads for the easily dispersed fuzzy seeds of cotton and even more so for very small seeds. Oilseed rape's seeds are small, about 200,000 per pound, and thus easily dispersed. That crop is not particularly well-domesticated. Rapeseed fruits can shatter (release) some seeds prior to and during harvest, allowing for the establishment of volunteers in and near the cultivated field. Alfalfa produces seeds of roughly the same size, its legumes shatter as easily as the siliques of rape. Cultivated creeping bentgrass cultivars are even less domesticated. They freely shatter their mature seeds. And those seeds are tiny, about ¼ of the size of alfalfa or rape seed. Feral transgenic papayas, typically on roadsides, may owe their establishment to seed dispersal, by birds or seeds thrown from the window of a speeding automobile (Manshardt et al., 2016). Three of the non-free-living long-term commercial transgenic crops—maize, soy, and squash—have large seeds and do not shatter. The fourth, sugar beet, is harvested before it sets seed.

Overall, outcrossing rate and pollen vector do not seem to play a particularly important role in discriminating among these groups. Both contain mostly outcrossing and mostly selfing species. On the other hand, some idiosyncrasies of mating system may be important: Among the stay-at-homes, soybean is the most highly self-fertilized of major commercialized transgenics, and sugar beet is the only crop that must be harvested prior to flowering. Those features may limit their ability to disperse a suitable number of seeds for colonization. For the other group, it is notable that oilseed rape and creeping bentgrass are known to be able to successful pollinate a mate at a distance in excess of a kilometer (Andersson and de Vicente, 2010).

The “virtual certainty” also seems more certain for certain transgenic traits. The vast majority of free-living populations that have been detected have been subject to very strong selection in favor of the trait based on the transgene. For example, prior to the introduction of virus-resistant papayas to the Hawai'ian Islands, the papaya crop and feral plants were in the process of extirpation there by the onslaught of the fatal papaya ringspot virus (Gonsalves, 2004). A gene for virus resistance would be strongly favored in that sort of environment. In contrast, virus-resistant squash has not established in free-living populations, and the viruses for which it is resistant are now known to typically play a minor role in regulating free-living populations (e.g., Quemada et al., 2008). In those environments, such resistance would confer only a minor advantage, if any (also, see Sasu et al., 2009).

The same logic holds for the abundant free-living populations bearing herbicide tolerance. Herbicide tolerant transgenes are favored in environments in which the selective herbicide is frequently used. We would expect glyphosate tolerance to be especially favored as glyphosate is “the dominant herbicide worldwide” (Duke and Powles, 2008). Indeed, only two of the 14 entries in Table 1 do not include glyphosate tolerance. Nonetheless, some caveats are appropriate. First, the abundance of herbicide-tolerance in free-living populations may be a simple correlate of the fact that it is, by far, the most abundant transgenic trait among the commercially grown varieties. Also, the trait is easily detected and noticed when a field is treated with the selective herbicide, revealing the tolerant survivors. Other traits, such as lepidopteran resistance, can only be identified with biochemical tools.

Of the 14 cases of free-living populations, a minority are problematic. Feral populations of multiple herbicide-tolerant transgenic Argentine oilseed rape have contributed to the rise of what is called “volunteer canola” as a significant weed in parts of Canada. Glyphosate tolerant oilseed rape also emerged as a problematic, if local, agronomic weed in the Buenos Aires province of Argentina. Likewise, glyphosate-tolerant creeping bentgrass has become a significant weed of irrigation canals in the US state of Oregon (National Research Council, 2017). The challenges of these problematic weeds are not insurmountable, alternate herbicides can be sought. But that solution is not always straightforward. These weeds have created headaches for farmers who must control them with alternate, less desirable, herbicides (Beckie et al., 2004). In the case of creeping bentgrass, only glyphosate was permitted by the US-Environmental Protection Agency for use as an herbicide in irrigation canals until 2017 when the agency approved a special local label for the use of glufosinate in irrigation canals in Oregon.

What can we learn from these examples about biosafety? With regards to the core principles of biosafety a comparison with traditionally improved plants is illuminating. Gene flow is the “exposure” component of traditional risk assessment's “exposure” x “hazard” = “risk” formulation. We see from the cases in Table 1 that crop plants already known to feralize or hybridize with free-living populations will do so with or without transgenes in their genomes. But with regard to the realized “hazard” component of the equation, the frequency of problems from free-living populations is somewhat greater than the experience with the feralization of traditionally improved crop plants (Ellstrand, 2003; Ellstrand et al., 2010). That is probably due to the fact that the problem plants have a transgenic phenotype for tolerance to novel herbicides. Notably, the problems are the result of the nature of the transgenic trait and not the result of transgenesis *per se*. Long ago, Ellstrand and Hoffman (1990) wrote, “The ecological impact of crop-weed hybridization will depend more on the biology of the crop, the wild relative, and the transferred gene than on the method of gene transfer.” While they did not anticipate the likelihood of direct feralization without hybridization, their emphasis on the biology of the entire system appears accurate.

Perhaps it's now worthwhile to attempt a first draft of a crude model for predicting whether novel alleles (created by any methodology) will establish themselves in free-living plants and, if so, under what circumstances they might contribute to the evolution of increased weediness or invasiveness. As Ellstrand and Hoffman (1990) suggest, let's concentrate on what we know about the biology of the system. Here are some factors that should contribute to the likelihood of novel allele establishment in free-living populations:

1. Crop species that are already known from feral (or wild) populations adjacent to field trials or cultivation. Pre-existing ferality may be a consequence of the following biological traits:
2. Seed dispersal appears to be an important component of the establishment of free-living populations, especially in the case of
  - Poorly domesticated crops that often shatter or otherwise disperse some of their seeds or fruits prior to harvest
  - Harvest-to-consumer supply chains that often result in some spillage of seed, grain, or fruit into the environment
  - The smaller the seed size, the more easily spontaneously dispersed.
3. Spontaneous pollen dispersal appears to play a secondary, but non-trivial, role, especially for those species capable of dispersing viable pollen sufficient distances to wild or otherwise free-living populations. Certainly, crops with the ability of successful pollen dispersal to one kilometer or more should receive close scrutiny, unless it is known that the crop will be fully harvested prior to flowering.
4. Perennial growth occurs at a much higher frequency among the free-living commercial transgenic crops relative to those that have not gone astray. It is not clear (at least to me) why this ecological factor is such a strong correlate.
5. The fitness effects of a transgenic trait as determined by its environment will play a role in its persistence and spread (Ellstrand, 2003):
  - Detrimental traits are expected to decrease in frequency over time unless replenished by repeated immigrant seed or pollen flow.
  - Neutral traits are expected to persist at the frequency that they are received.
  - Beneficial traits are expected to increase in frequency and spread.
  - Evaluating the selective value of a trait may be challenging. For example, as detailed above, the trait “virus resistance” was beneficial for Hawaiian feral papayas in their specific environment but not for free-living squashes in the United States.

Although determining the fitness effects of a novel trait may be challenging, much of the relevant biological data regarding the crop and its wild and weedy relatives should not difficult to obtain. For example Andersson and de Vicente (2010) book is a good start to evaluate the world's most important crops for details of the seed and pollen dispersal as well as what is known about their feral and wild relatives. Because of the recent research interest on the topic any evaluation should be supplemented with an online literature search. A similar, systematic and structured approach, has been utilized as part of recent environmental risk assessments of upcoming transgenic African crops intended to be grown near related free-living populations (Hokanson et al., 2010, 2016; Huesing et al., 2011).

I finish with a thought-experiment. Which existing transgenic species will be the next to join those in Table 1? Table 2 lists some possible candidates.

**Table 2:** Potential candidates for as-yet undiscovered free-living transgenic plant populations.

**Species (common name)**	**Transgenic trait(s)**
*Arabidopsis thaliana* (thale cress)	Numerous and varied
*Festuca arundinacaea* (tall fescue)	Glyphosate tolerance/ Enhanced turfgrass quality
*Paspalum notatum* (bahiagrass)	ALS-inhibiting herbicide tolerance
*Poa pratensis* (Kentucky bluegrass)	Glyphosate tolerance/ Enhanced turfgrass quality
*Populus nigra* (black poplar)^a^	Lepidopteran resistance
*Solanum melogena* (eggplant)	Lepidopteran resistance
*Stenotaphrum secundatum* (St. Augustine grass)	Glyphosate tolerance/ Enhanced turfgrass quality

a *anecdotal free-living transgenic populations (Bauer-Panskus et al., 2013)*.

All but *Arabidopsis thaliana* have been approved for environmental release in at least one country. *A. thaliana* was chosen for the list because it has been the primary model organism for transgenic research for decades. It has been used as a research organism at hundreds, if not thousands, of colleges, universities, and other research entities. Dozens of field trials have been authorized. Native to the Old World, wild populations have colonized disturbed habitats of pan-temperate regions globally. An adult plant is capable of producing hundreds of tiny seeds (< 0.5 mm diameter). The species is largely self-pollinating with opportunity for insect-pollination. The overwhelming global scientific use of this plant suggests that seed spillage might have established volunteer or feral populations of transgenic plants somewhere in the world. If so, they would most likely be in human-disturbed habitats near to where research on the species is conducted.

The rest of Table 2's candidate species have transgenic varieties that are either in cultivation or have been authorized or cultivation. Non-transgenic versions of those species are known to exist in persistent feral or wild populations. All but *Solanum melogena* (brinjal/eggplant) have easily dispersed propagules. *Populus nigra* produces plumed seeds. The rest are grass species with small to very small (especially *Poa pratensis*) caryopses. The grass species are all wind-pollinated but their maximum viable pollen dispersal distances are unknown. *Populus nigra* is dioecious and insect-pollinated. *S. melogena* is largely self-pollinating with opportunity for insect-pollination. All are perennial, and all but *S. melogena* are capable of vigorous vegetative reproduction. Whether their associated transgenic traits confer any fitness advantage depends a lot on the environment in which the free-living populations grow. The abundance of herbicide tolerant cases in Table 1 suggests that the glyphosate tolerant species in Table 2 might have an advantage if they disperse into unintended areas in which that herbicide is commonly used. Unless additional confinement features are utilized for these species (e.g., some *Stenophorum secundatum* cultivars are seed sterile), given sufficient cultivation area and sufficient time, taken as a group, it is likely that at least one will donate its transgenes to a free-living population. But, given the foregoing examples, it might take decades for that prediction to be realized.

## Author contributions

The author confirms being the sole contributor of this work and approved it for publication.

### Conflict of interest statement

The author declares that the research was conducted in the absence of any commercial or financial relationships that could be construed as a potential conflict of interest.

## References

[B1] Andersson M. de Vicente M. C. (2010). Gene Flow Between Crops and Their Wild Relatives. Baltimore, MD: Johns Hopkins University Press.

[B2] Bashandy H. Teeri T. (2017). Genetically engineered petunias on the market. Planta 246, 277–280. 10.1007/s00425-017-2722-8 28647812 PMC5522655

[B3] Bauer-Panskus A. Breckling B. Hamberger S. Then C. (2013). Cultivation-independent establishment of genetically engineered plants in natural populations: current evidence and implications for EU regulation. Environ. Sci. Eur. 25:34 10.1186/2190-4715-25-34

[B4] Beckie H. J. Séguin-Swartz G. Nair H. Warwick S. I. Johnson E. (2004). Multiple herbicide-resistant canola can be controlled by alternate herbicides. Weed Sci. 52, 152–157. 10.1614/P2002-163

[B5] Booker H. M. Mischkolz J. M. St. Louis M. Lamb E. G. (2014). Analysis of the prevalence of CDC Triffid transgenic flax in Canadian seed stocks. AgBioforum 17, 75–83.

[B6] Busi R. Powles S. B. (2016). Transgenic glyphosate-resistant canola (*Brassica napus*) can persist outside agricultural fields in Australia. Agric. Ecosyst. Environ. 22, 28–34. 10.1016/j.agee.2015.12.028

[B7] Clapp J. (2008). Illegal GMO releases and corporate responsibility: questioning the effectiveness of voluntary measures *Ecol*. Econ. 66, 348–358. 10.1016/j.ecolecon.2007.09.006

[B8] Colwell R. E. Norse E. A. Pimentel D. Sharples F. E. Simberloff D. (1985). Genetic engineering in agriculture. Science 229, 111–112. 10.1126/science.229.4709.111 17746269

[B9] Council for Agricultural Science and Technology (2007). Implications of Gene Flow in the Scale-Up and Commercial Use of Biotechnology-Derived Crops: Economic and Policy Considerations. Issue Paper 37. Ames, IA: Council for Agricultural Science and Technology.

[B10] Daroit D. Nascimento L. F. (2009). The influence of the actor network on the innovative process of transgenic soybean in Rio Grande Do Sul, Brazil. J. Technol. Manage. Innov. 4, 150–160. 10.4067/S0718-27242009000400013

[B11] Demeke T. Perry D. J. Scowcroft W. R. (2006). Adventitious presence of GMOs: scientific overview for Canadian grains. Can. J. Plant Sci. 86, 1–23. 10.4141/P05-114

[B12] Devos Y. Demont M. Dillen K. Reheul D. Kaiser M. Sanvido O. (2009). Coexistence of genetically modified (GM) and non-GM crops in the European Union. Rev. Agron. Sustain. Dev. 29, 11–30. 10.1051/agro:2008051

[B13] Duke S. O. Powles S. B. (2008). Glyphosate: a once-in-a-century herbicide. Pest Manage. Sci. 64, 319–325. 10.1002/ps.1518 18273882

[B14] Dyer G. A. Serratos-Hernández J. A. Perales H. R. Gepts P. Piñeyro-Nelson A. Chávez A.. (2009). Dispersal of transgenes through maize seed systems in Mexico. PLoS ONE 4:e5734. 10.1371/journal.pone.0005734 19503610 PMC2685455

[B15] Ellstrand N. C. (2003). Dangerous Liaisons? When Cultivated Plants Mate with their Wild Relatives. Baltimore, MD: Johns Hopkins University Press.

[B16] Ellstrand N. C. (2012). Over a decade of transgenes out of place, in Regulation of Agricultural Biotechnology: The United States and Canada, eds Wozniak C. A. McHughen A. (Dordrecht: Springer), 123–135.

[B17] Ellstrand N. C. Hoffman C. A. (1990). Hybridization as an avenue for escape of engineered genes. Bioscience 40, 438–442. 10.2307/1311390

[B18] Ellstrand N. C. Heredia S. M. Leak-Garcia J. A. Heraty J. M. Burger J. C. Yao L.. (2010). Crops gone wild: evolution of weeds and invasives from domesticated ancestors. Evol. Appl. 3, 494–504. 10.1111/j.1752-4571.2010.00140.x 25567942 PMC3352506

[B19] Gonsalves D. (2004). Transgenic papaya in Hawaii and beyond. AgBioforum 7, 36–40.

[B20] Goodman R. M. Newell N. (1985). Genetic engineering of plants for herbicide resistance: status and prospects, in Engineered Organisms in the Environment: Scientific Issues, eds Halvorson H. O. Pramer D. Rogul M. (Washington, DC: American Society for Microbiology), 47–53.

[B21] Greene S. L. Kesoju S. R. Martin R. C. Kramer M. (2015). Occurrence of transgenic feral alfalfa (*Medicago sativa* subsp. *sativa L.)* in alfalfa seed production areas in the United States. PLoS ONE 10:e0143296. 10.1371/journal.pone.0143296 26699337 PMC4689365

[B22] Hall L. Topinka K. Huffman J. Davis L. Allen A. (2000). Pollen flow between herbicide-resistant *Brassica napus* is the cause of multiple-resistant *B. napus* volunteers. Weed Sci. 48, 688–694. 10.1614/0043-1745(2000)048[0688:PFBHRB]2.0.CO;2

[B23] Han S. M. Kim D. Y. Uddin M. R. Hwang K. S. Lee B. Kim C.-G. (2014). Appearance/instance of genetically modified maize at grain receiving harbors and along transportation routes in Korea. Weed Turfgrass Sci. 3, 221–224. 10.5660/WTS.2014.3.3.221

[B24] Harper J. H. (1977). Population Biology of Plants. London: Academic Press.

[B25] Hecht M. Oehen B. Schulze J. Brodmann P. Bagutti C. (2014). Detection of feral GT73 transgenic oil-seed rape (*Brassica napus*) along railway lines on entry routes to oilseed factories in Switzerland. Environ. Sci. Pollut. Res. 21, 1455–1465. 10.1007/s11356-013-1881-9 23917737

[B26] Hokanson K. E. Ellstrand N. C. Dixon A. G. O. Kulembeka H. P. Olsen K. M. Raybould A. (2016). Risk assessment of gene flow from genetically engineered virus resistant cassava to wild relatives in Africa: an expert panel report. Transgenic Res. 25, 71–81. 10.1007/s11248-015-9923-3 26667472

[B27] Hokanson K. E. Ellstrand N. C. Ouedraogo J. T. Olweny P. A. Schaal B. A. Raybould A. F. (2010). Biofortified sorghum in Africa: using problem formulation to inform risk assessment. Nat. Biotechnol. 28, 900–903. 10.1038/nbt0910-900 20829822

[B28] Huesing J. Romeis J. Ellstrand N. Raybould A. Hellmich R. Wolt J.. (2011). Regulatory considerations surrounding the deployment of Bt-expressing cowpea in Africa: report of the deliberations of an expert panel. GM Crops Food 2, 211–224. 10.4161/gmcr.2.3.18689 22179194

[B29] Katsuta M. Matsuo K. Yoshimura Y. Ohsawa R. (2015). Long-term monitoring of feral genetically modified herbicide-tolerant *Brassica napus* populations around unloading Japanese ports. Breed. Sci. 65, 265–275. 10.1270/jsbbs.65.265 26175624 PMC4482177

[B30] Kelly A. F. George R. A. T. (1998). Encyclopaedia of Seed Production of World Crops. Chicester: Wiley.

[B31] Kershen D. L. McHughen A. (2005). Adventitious Presence. CAST Commentary. QTA2005-1. Ames, IA: Council for Agricultural Science and Technology.

[B32] Knispel A. L. McLachlan S. M. (2010). Landscape distribution and persistence of genetically modified oilseed rape (*Brassica napus*) in Manitoba, Canada. Environ. Sci. Pollut. Res. 17, 13–25. 10.1007/s11356-009-0219-0 19588180

[B33] Knispel A. L. McLachlan S. M. Van Acker R. C. Friesen L. F. (2008). Gene flow and multiple herbicide resistance in escaped canola populations. Weed Sci. 56, 72–80. 10.1614/WS-07-097.1

[B34] Ledford H. (2007). Out of bounds. Nature 445, 132–133. 10.1038/445132a 17215811

[B35] Lee B. Kim C.-G. Park J.-Y. Park K. W. Kim H.-J. Yi H. (2009). Monitoring the occurrence of genetically modified soybean and maize in cultivated fields and along the transportation routes of the Incheon Port in South Korea. Food Control 20, 250–254. 10.1016/j.foodcont.2008.05.006

[B36] Manshardt R. M. Mello C. L. Lum S. D. Ta L. (2007). Tracking papaya pollen movement with the GUS transgene marker. Acta Hortic. 740, 183–187. 10.17660/ActaHortic.2007.740.21

[B37] Manshardt R. Bishaw D. Pilz K. Stewart C. N. (2016). Gene flow from commercial transgenic papaya fields into feral populations in Hawai'i. Acta Hortic. 1124, 33–40. 10.17660/ActaHortic.2016.1124.5

[B38] Marvier M. Van Acker R. C. (2005). Can crop transgenes be kept on a leash? Front. Ecol. Environ. 3, 99–106. 10.1890/1540-9295(2005)003[0093:CCTBKO]2.0.CO;2

[B39] Merotto A. Goulart I. C. G. R. Nunes A. L. Kalsing A. Markus C. Menezes V. G. (2016). Evolutionary and social consequences of introgression of NON-transgenic herbicide resistance from rice to weedy rice in Brazil. Evol. Appl. 9, 837–846. 10.1111/eva.12387 27468302 PMC4947146

[B40] Munier D. J. Brittan K. Lanini W. T. (2012). Seed bank persistence of genetically modified canola in California. Environ. Sci. Pollut. Res. 19, 2281–2284. 10.1007/s11356-011-0733-8 22258428

[B41] National Research Council (1989). Field Testing Genetically Modified Organisms: Framework for Decisions. Washington, DC: National Academy Press. 25144044

[B42] National Research Council (2002). Environmental Effects of Transgenic Plants. Washington, DC: National Academy Press

[B43] National Research Council (2004). Biological Confinement of Genetically Engineered Organisms. Washington, DC: National Academy Press.

[B44] National Research Council (2017). Genetically Engineered Crops. Washington, DC: National Academy Press.

[B45] OECD (2018). OECD Seed Schemes (2018). Paris: Organization for Economic Co-operation and Development Available online at http://www.oecd.org/tad/code/oecd-seed-schemes.pdf

[B46] Owen M. D. K. (2005). Maize and soybeans—controllable volunteerism without ferality? in Crop Ferality and Volunteerism, ed Gressel J. (Boca Raton, FL: CRC Press), 209–30.

[B47] Pandolfo C. E. Presotto A. Carbonell F. T. Ureta S. Poverne M. Cantamutto M. (2016). Transgenic glyphosate-resistant oilseed rape (*Brassica napus*) as an invasive weed in Argentina*. Environ. Sci. Pollut. Res*. Int. 23, 24081–24091. 10.1007/s11356-016-7670-5 27638808

[B48] Pandolfo C. E. Presotto A. Carbonell F. T. Ureta S. Poverne M. Cantamutto M. (2018). Transgene escape and persistence in an agroecosystem: the case of glyphosate-resistant *Brassica rapa* L.in central Argentina. Environ. Sci. Pollut. Res. Int. 25, 6251–6264. 10.1007/s11356-017-0726-3 29243152

[B49] Park K. W. Lee B. Kim C.-G. Kim D. Y. Park J.-Y. Ko E.-M. (2009). Monitoring the occurrence of genetically modified maize at a grain receiving port and along transportation routes in the Republic of Korea. Food Control 21, 456–461. 10.1016/j.foodcont.2009.07.006

[B50] Prendeville H. R. Ye X. Morris T. J. Pilson D. (2012). Virus infections in wild plant populations are both frequent and often unapparent. Am. J. Bot. 99, 1033–1042. 10.3732/ajb.1100509 22645099

[B51] Price B. Cotter J. (2014). The GM contamination register: a review of recorded contamination incidents associated with genetically modified organisms (GMOs), 1997–2013. Int. J. Food Contam.1:5 10.1186/s40550-014-0005-8

[B52] Quemada H.L. Strehlow D. S. Decker-Walters Staub J. E. (2008). Population size and incidence of virus infection in free-living populations of *Cucurbita pepo*. Environ. Biosafety Res. 7, 185–196. 10.1051/ebr:2008022 19081007

[B53] Ryffel G. U. (2014). Transgene flow: facts, speculations and possible countermeasures. GM Crops Food 5, 249–258. 10.4161/21645698.2014.945883 25523171 PMC5033179

[B54] Sasu M. A. Ferrari M. J. Du D. Winsor J. A. Stephenson A. G. (2009). Indirect costs of a nontarget pathogen mitigate the direct benefits of a virus-resistant transgene in wild Cucurbita. Proc. Natl. Acad. Sci. U.S.A. 106, 19067–19071. 10.1073/pnas.0905106106 19858473 PMC2776422

[B55] Schafer M. G. Ross A. A. Londo J. P. Burdick C. A. Lee E. H. Travers S. E.. (2011). The establishment of genetically engineered canola populations in the U. S. PLoS ONE 6:e25736. 10.1371/journal.pone.0025736 21998689 PMC3187797

[B56] Smyth S. J. Kerr W. A. Phillips P. W. B. (2017). Global trade impacts from low level presence. *Biotech*. Regul. Trade 51, 55–73. 10.1007/978-3-319-53295-0_4

[B57] Stein A. J. Rodríguez-Cerezo E. (2010). Low-level presence of new GM crops: an issue on the rise for countries where they lack approval. AgBioforum 13, 173–182.

[B58] Strayer D. (2002). Identity-Preserved Systems: A Reference Handbook. Boca Raton, FL: CRC Press.

[B59] University of California Davis (2003). Tomato Seed from Seed Bank Found to be Genetically Modified. UC Davis News and Information. Available online at https://www.ucdavis.edu/news/tomato-seed-seed-bank-found-be-genetically-modified/.

[B60] USDA (2007). Report of LibertyLink Rice Incidents. Available online at https://www.foeeurope.org/sites/default/files/press_releases/ricereport10-2007.pdf.

[B61] USDA (2014). Glyphosate-Resistant Wheat. Available online at https://www.aphis.usda.gov/aphis/ourfocus/biotechnology/brs-news-and-information/ct_news_wheat.

[B62] USDA (2015). Justification for Moving ge Wheat Field Trials to Permit. Available online at https://www.aphis.usda.gov/biotechnology/downloads/wheat_permit_change.pdf.

[B63] USDA (2016). Update Regarding Detection of GE Wheat Volunteer Plants in Washington State. Available online at https://www.aphis.usda.gov/aphis/ourfocus/biotechnology/brs-news-and-information/wheat_fact_finding_closed.

[B64] USDA (2017). Unauthorized genetically engineered petunias. Available online at https://www.aphis.usda.gov/aphis/ourfocus/biotechnology/brs-news-and-information/petunia_overview.

[B65] Warwick S. I. Légère A. Simard M.-J. James T. J. (2008). Do escaped transgenes persist in nature? the case of an herbicide resistance transgene in a weedy Brassica rapa population. Mol. Ecol. 17, 1387–1395. 10.1111/j.1365-294X.2007.03567.x 17971090

[B66] Warwick S. I. Simard M.-J. Légère A. Beckie H. J. Braun L. Zhu B.. (2003). Hybridization between transgenic *Brassica napus* L. and its wild relatives: *Brassica rapa* L*., Raphanus raphanistrum* L*., Sinapis arvensis* L. *and Erucastrum gallicum* (Willd.) O. E. Schulz. Theor. Appl. Genet. 107, 528–529. 10.1007/s00122-003-1278-0 12721639

[B67] Wegier A. Piñeyro-Nelson A. Alarcon J. Gálvez-Mariscal A. Álvarez-Buylla E. R. Piñero D. (2011). Recent long-distance transgene flow into wild populations conforms to historical patterns of gene flow in cotton (*Gossypium hirsutum*). Mol. Ecol. 20, 4182–4194. 10.1111/j.1365-294X.2011.05258.x 21899621

[B68] Yoshimura Y. Beckie H. Matsuo K. (2006). Transgenic oilseed rape along transportation routes and port of Vancouver in western Canada. Environ. Biosafety Res. 5, 67–75. 10.1051/ebr:2006019 17328853

[B69] Zapiola M. L. Campbell C. K. Butler M. D. Mallory-Smith C. A. (2008). Escape and establishment of transgenic glyphosate-resistant creeping bentgrass (*Agrostis stolonifera*) in Oregon, USA: a 4-year study. J. Appl. Ecol. 45, 486–494. 10.1111/j.1365-2664.2007.01430.x

[B70] Zapiola M. L. Mallory-Smith C. A. (2012). Crossing the divide: gene flow produces intergeneric hybrid in feral transgenic creeping bentgrass population. Molec. Ecol. 21, 4672–4680. 10.1111/j.1365-294X.2012.05627.x 22625177

[B71] Zapiola M. L. Mallory-Smith C. A. (2017). Pollen-mediated gene flow from transgenic perennial creeping bentgrass and hybridization at the landscape level. PLoS ONE 12:e0173308. 10.1371/journal.pone.0173308 28257488 PMC5336273

